# The Soft-Strain Effect Enabled High-Performance Flexible Pressure Sensor and Its Application in Monitoring Pulse Waves

**DOI:** 10.34133/research.0002

**Published:** 2022-12-15

**Authors:** Yue Li, Yuan Wei, Yabao Yang, Lu Zheng, Lei Luo, Jiuwei Gao, Hanjun Jiang, Juncai Song, Manzhang Xu, Xuewen Wang, Wei Huang

**Affiliations:** ^1^ Frontiers Science Center for Flexible Electronics (FSCFE) and Institute of Flexible Electronics (IFE), Northwestern Polytechnical University, Xi’an, 710072, China.; ^2^ MIIT Key Laboratory of Flexible Electronics (KLoFE), Northwestern Polytechnical University, Xi’an, 710072, China.; ^3^ Shaanxi Key Laboratory of Flexible Electronics (KLoFE), Northwestern Polytechnical University, Xi’an, 710072, China.; ^4^ Key Laboratory of Flexible Electronics of Zhejiang Province, Ningbo Institute of Northwestern Polytechnical University, 218 Qingyi Road, Ningbo, 315103, China.; ^5^ State Key Laboratory of Organic Electronics and Information Displays, Institute of Advanced Materials (IAM), Nanjing University of Posts and Telecommunications, Nanjing, 210023, China.; ^6^ Key Laboratory of Flexible Electronics (KLoFE) and Institute of Advanced Materials (IAM), Nanjing Tech University (NanjingTech), Nanjing, 211800, China.

## Abstract

Flexible and wearable pressure sensors attached to human skin are effective and convenient in accurate and real-time tracking of various physiological signals for disease diagnosis and health assessment. Conventional flexible pressure sensors are constructed using compressible dielectric or conductive layers, which are electrically sensitive to external mechanical stimulation. However, saturated deformation under large compression significantly restrains the detection range and sensitivity of such sensors. Here, we report a novel type of flexible pressure sensor to overcome the compression saturation of the sensing layer by soft-strain effect, enabling an ultra-high sensitivity of ~636 kPa^−1^ and a wide detection range from 0.1 kPa to 56 kPa. In addition, the cyclic loading-unloading test reveals the excellent stability of the sensor, which maintains its signal detection after 10,000 cycles of 10 kPa compression. The sensor is capable of monitoring arterial pulse waves from both deep tissue and distal parts, such as digital arteries and dorsal pedal arteries, which can be used for blood pressure estimation by pulse transit time at the same artery branch.

## Introduction

Flexible electronics are of significant importance in promoting the information age’s evolution toward an intelligent future era, especially for disruptive medical technology. As a crucial unit, flexible pressure sensors are extensively studied because of their potential applications in clinic treatments [1–3], electronic skins [4–6], healthcare monitors [7–10], and human-machine interfaces [11–13]. Up to now, various configurations are developed for the realization of pressure sensors, which include piezoresistive, piezoelectric, capacitive, and field effect transistor types. These sensors are expected to have a broad linear detection range with high sensitivity for multifunctional scenarios. Among them, piezoresistive sensors are widely used because of their simple configuration [14], decent stability [15], high sensitivity [16], wide detection range [17], easy fabrication [18], and diverse structures [19]. The working principle for piezoresistive sensors is based on the resistance change caused by the deformation of sensing materials. When pressure is applied, the sensing layer is compressed, resulting in a gradual saturation of deformation because of the concentrated internal stress. Therefore, the sensitivity of such sensors gradually decreases with the increasing pressure [20], which restrains the applications in saturated region. Similarly, capacitive pressure sensors have the same behavior due to the limited deformation of the dielectric layer [21–24]. Despite various strategies in improving the sensitivity of sensors [25–28], however, it is a challenge to retain high sensitivity in pressure detection range.

Recently, flexible pressure sensors are utilized in real-time tracking pulse waves for health monitoring and assessment because pulse waves contain health-related physiological signals [29–32]. Typically, heart rate, respiratory rhythm, and blood pressure can be measured by monitoring pulse waves. Percussion wave (P), tidal wave (T), and dicrotic wave (D) in pulse signals, as well as radial augmentation index and auxiliary blood pressure index (*k*), are useful in evaluating the risk of cardiovascular diseases and arteriosclerosis [33,34]. The interface between flexible pressure sensors and human skin is the key to obtain high-quality (stable and reliable) signals, which requires a sensor with high sensitivity for the detection of tiny pulse waves. Typically, a preloaded external force is employed to enhance the contact interface [35,36]. However, the pulse waves are susceptible to preloaded forces when the sensor is wrapped by a band on human skin. Therefore, it is highly desirable to develop a flexible pressure sensor that has high sensitivity in detecting pulses under external forces.

Herein, we report on a high-performance flexible pressure sensor that uses the soft-strain effect with an optimized multilayer structure. Our flexible pressure sensor shows a positive resistance response caused by tensile strain of the conductive film, and its sensitivity increases with pressure, which is capable of detecting large external forces. A microsphere is employed to stick on the top electrode to improve the sensitivity by amplifying the strain caused by external pressure. The sensor exhibits high sensitivity (~636 kPa^−1^), excellent stability (10,000 cycles under 10 kPa), fast response time, and a broad detection range (0.1 to 56 kPa). We further demonstrate its applications in tracking pulse waves in real time for monitoring blood pressure. The pulse signals are collected from radial artery, distal artery, dorsal pedal artery, and first dorsal metatarsal artery. More importantly, a flexible sensor array has been fabricated to detect the pulse distribution for early diagnosis of arteriosclerosis.

## Results

### Fabrication and simulation of flexible pressure sensors

**Fig. 1.**
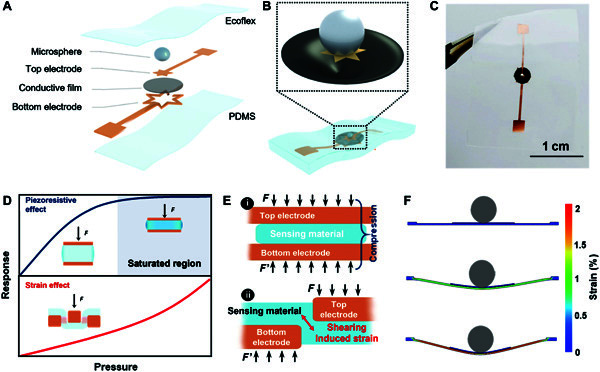
Simulation and realization of the soft-strain effect based pressure sensors. (A) Schematic illustration of the sensor, comprising Ecoflex encapsulation, tin microstructure, copper foil electrodes, carbon oil film, and polydimethylsiloxane (PDMS) substrate. (B) The soft-strain effect sensing unit design. (C) A photograph of the pressure-sensing unit. (D) The different trends of resistance change of piezoresistive sensors and the soft-strain effect sensors. (E) A comparison of sensing mechanisms of (i) piezoresistive sensors and (ii) the soft-strain effect sensors. (F) Finite element analysis of the sensing unit with the same proportion of the real structure.

As shown in Fig. 1A-C, the proposed pressure sensor comprises several parts: conductive carbon oil film, microsphere top electrode, ring bottom electrode, polydimethylsiloxane (PDMS) substrate, and Ecoflex encapsulation (Fig. 1A). The conductive film is pressed downward through the hollow of the bottom electrode when pressure is applied to the top microsphere, generating a microscopic radial strain instead of compressed deformation (Fig. 1B and Fig. S1). The pressure is evaluated through resistance changes caused by the stretching of the conductive film. The sensitivity in resistance response can be calculated by Eq. 1:

$$S=\frac{\left(R-R_{0}\right)/R_{0}}{\Delta P}$$ (1)

where *S* is defined as sensitivity, ∆*P* is the change in pressure, *R*_0_ is the initial resistance, and *R* is the resistance under a certain pressure. The typical sandwich piezoresistive structure contains 3 parts: the top electrode, the sensing layer, and the bottom electrode. When pressure is applied to the surface of the typical piezoresistive sensor, the elastic dielectric is compressed, resulting in a decreased resistance. The piezoresistive sensing materials also have Poisson expansion during compression (Fig. S2A,B). It differs from the soft tensile strain that stretched sensing layers in the longitudinal, avoiding the compression transversely and the accumulation of internal stress (Fig. S2E) [37]. The hardening of structures and changes in resistance of the piezoresistive sensor during compression tend toward 100%, significantly limiting a high resolution. The issue can be resolved by converting the piezoresistive effect into the soft-strain effect. The introduction of strain structures is practical that embeds beams into elastic material, and pressure can be detected by measuring the strain of cantilevers [38,39]. But the beam strain leads to continuously increasing positive resistance values that are linear with pressure in a narrow range [40]. In addition, a regular micromechanical system normally has rigid cantilever beams integrated into an elastic substrate that inhibits flexible deformation [41]. Considering the structural and intrinsic flexibility, a conductive film is suitable for soft-strain generation. Young’s modulus and size differences between layers in the strain-type structure make the conductive film generate stretching strain. Thus, the changes in resistance value are positive. The increasing resistance with pressure is different from piezoresistive type structures, avoiding compression saturation (Fig. 1D).

The novel soft-strain structures are fabricated in several steps (Fig. S3A). Firstly, direct laser writing has been used to manufacture the microelectrodes on a 6-μm-thick copper foil, followed by secondary transfer, ultrasonic cleaning, and drying (Fig. S3B). Next, we use the direct laser to pattern polyimide (PI) mask. Then, conductive carbon oil is spin-coated over the PI patterned mask to form a conductive carbon film. Note that the PI mask is peeled off before the film is completely dried (Fig. S3B). Subsequently, the soft-strain structure is completed by assembling the top electrode and a tin microsphere at the film center. Finally, Ecoflex is utilized to encapsulate the structure to obtain the sensor. After baking, strong bonds are formed between the multilayers.

A 2-dimensional implicit dynamic finite element analysis (FEA) has been performed to investigate the working mechanisms. The effectiveness of the soft-strain structure is evidenced by the FEA simulation (Fig. S2C). In the simulation, the sensing layer is fixed between the 2 electrodes. The bottom electrode is bonded to the PDMS substrate, and the sensing film is stuck to the bottom electrode. The microsphere and top electrode placed in the center of the sensing film are bonded together using Ecoflex. Specifically, when pressure is applied to the surface, the microsphere and top electrode push the conductive film down through the hollow of the bottom electrode to generate mechanical strain (Fig. 1E). As shown in Fig. S4, in situ observation for the cross-section of the microstructure under different pressure also suggests that soft-strain structures successfully convert the compression of sensing materials to stretching strain. Note that, in our simulation, there is no slip between the top electrode and sensing materials. In addition, the materials are homogenous, and the thermal effect is neglected.

We also simulate the stretching strain under different pressure (Fig. 1F). Initially, no strain is produced without pressure. Interestingly, the conductive film conformal to the elastic substrate is stretched when the applied pressure increases (Fig. 1F). The generated strain firstly concentrates at the interfaces of the microsphere and elastic conductive film. Then, the strain gradually concentrates on the interfacial contact points between the metal electrodes and elastic conductive film as pressure increases (Fig. S2D). From the simulation results, we can see that the microsphere works as a strain amplifier. The interfaces between the bottom electrode and the conductive film determine the stretching strain intensity. Then, we design a complex shape of the bottom electrode to introduce more strain-sensitive points.

### High performance in the detection of pressure

**Fig. 2.**
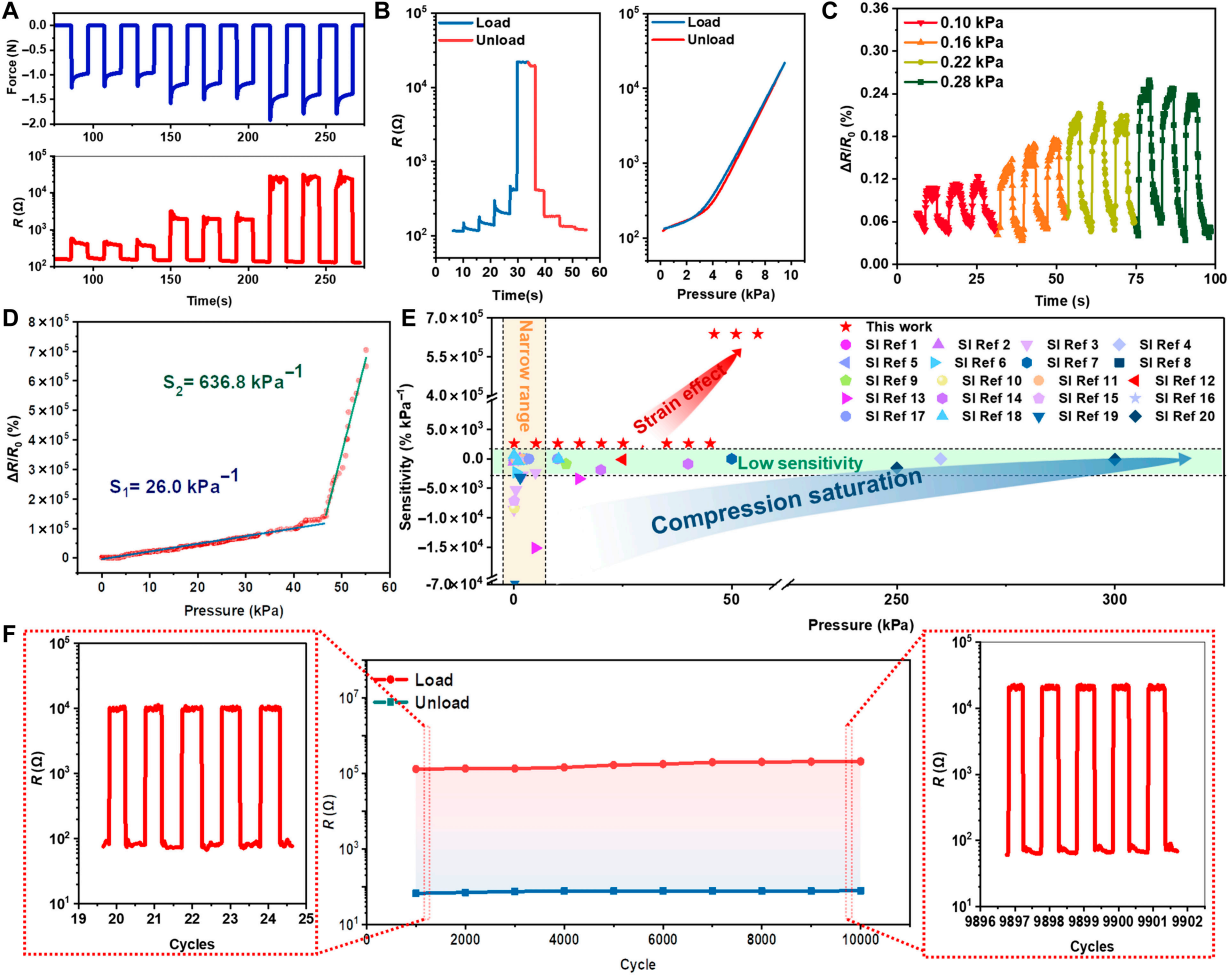
Sensing performance of the flexible pressure sensor. (A) The resistance response of the sensor under force. (B) The sensor’s static and dynamic responses to stepwise pressure loading-unloading from 0.1 kPa to 10 kPa. (C) The resistance variation under ultra-low pressures. (D) The resistance change as a function of pressure from 0.1 kPa to 56 kPa. (E) Summary of the sensitivity for various flexible pressure sensors. (F) Reliability test over 10,000 cycles under 10 kPa.

We then construct a testing system to evaluate the performance of the pressure sensor. A stepper motor with a force gauge provides periodic compressive loads as input, and the output resistance values are recorded using a high-precision source meter. As shown in Fig. 2A, the flexible sensor exhibits positive resistance responses within the mechanical detection limit. We also investigate stepped resistance changes by applying stepped force, indicating excellent pressure resolution. The pressure sensor also has great dynamic mechanical performance. The “shoulder peak” of the force signals corresponds to the “shoulder peak” of the resistance signals (Fig. S5B). Furthermore, the stepwise and rapid pressure repeats loading and unloading (from 0 kPa to 12 kPa) for several cycles (Fig. 2B and Fig. S6). Our sensor exhibits good pressure resolution and mechanical reliability. Figure 2C and Fig. S7 show the capability to detect the ultra-lightweight in a low-pressure scale (from 0.1 kPa to 0.28 kPa), making the precise acquisition of signals below 1 kPa. Our sensor can successfully monitor 0.3 kPa (Fig. S7). Because of the sensor’s ultralow detection limitation and high pressure resolution, the sensor precisely records dynamic loading and unloading under several cycles.

The sensitivity and linear detection range are studied by recording the changes in the resistance as a function of pressure. Figure 2D illustrates the relationship between the pressure and resistance of the sensor. It can be calculated from Eq. 1 that the sensitivity is 26 kPa^−1^ from 0.1 kPa to 46 kPa, with a small nonlinearity error (*R*_1_^2^ = 0.978 and *R*_2_^2^ = 0.968). Further, when the pressure increases from 46 kPa to 56 kPa, the stress gradually concentrates at the interfaces between the electrodes and conductive film because of differences in mechanical properties. The sensitivity increases to 636.8 kPa^−1^. We attribute the increased sensitivity to the tensile strain of ultrathin conductive film under downward pressure (Fig. S8). In addition to the applied pressure, the shapes of interfaces between the conductive film and the metal electrodes also have a significant influence on the stress concentration (Fig. S9). As a result, the sensitivity increases with larger interfaces between the conductive film and the metal electrodes. Compared to literature, our work provides a novel way to solve the compression saturation problem of sensing material, as shown in Fig. 2E and Table S1).

Subsequently, the reliability of the sensor is tested by applying cyclic loading-unloading with a pressure of 10 kPa. As shown in Fig. 2F, no obvious variation and fluctuation in resistance are observed for 10,000 cycles. The high durability is attributed to the strong interfacial bond between the electrodes and the conductive film. The conformal package coating of liquid PDMS maximizes the bond strength of layers. Moreover, the response time is also tested. As shown in Fig. S5A, the response time and recovery time are approximately 130 and 160 ms, respectively.

### Applications in monitoring the pulse waves

We apply the flexible pressure sensor to measure physiological signals according to the linear pressure detection range (0.1 to 12 kPa). Arteries are distributed in the human body. The characteristic of arterial pulse waves is a vital reference for the latent value information of cardiovascular disease. Nevertheless, arterial pulse waves are weak signals that are susceptible to noise caused by body movement, muscle and nervous tension, breath, etc. Thus, only superficial arteries, such as the radial, ulnar, carotid, and femoral artery, can be detected by most pressure sensors. In contrast, the signals from peripheral arterials are often overlooked because of insufficient measurement accuracy, despite that they contain important potential factors for early cardiovascular disease screening.

**Fig. 3.**
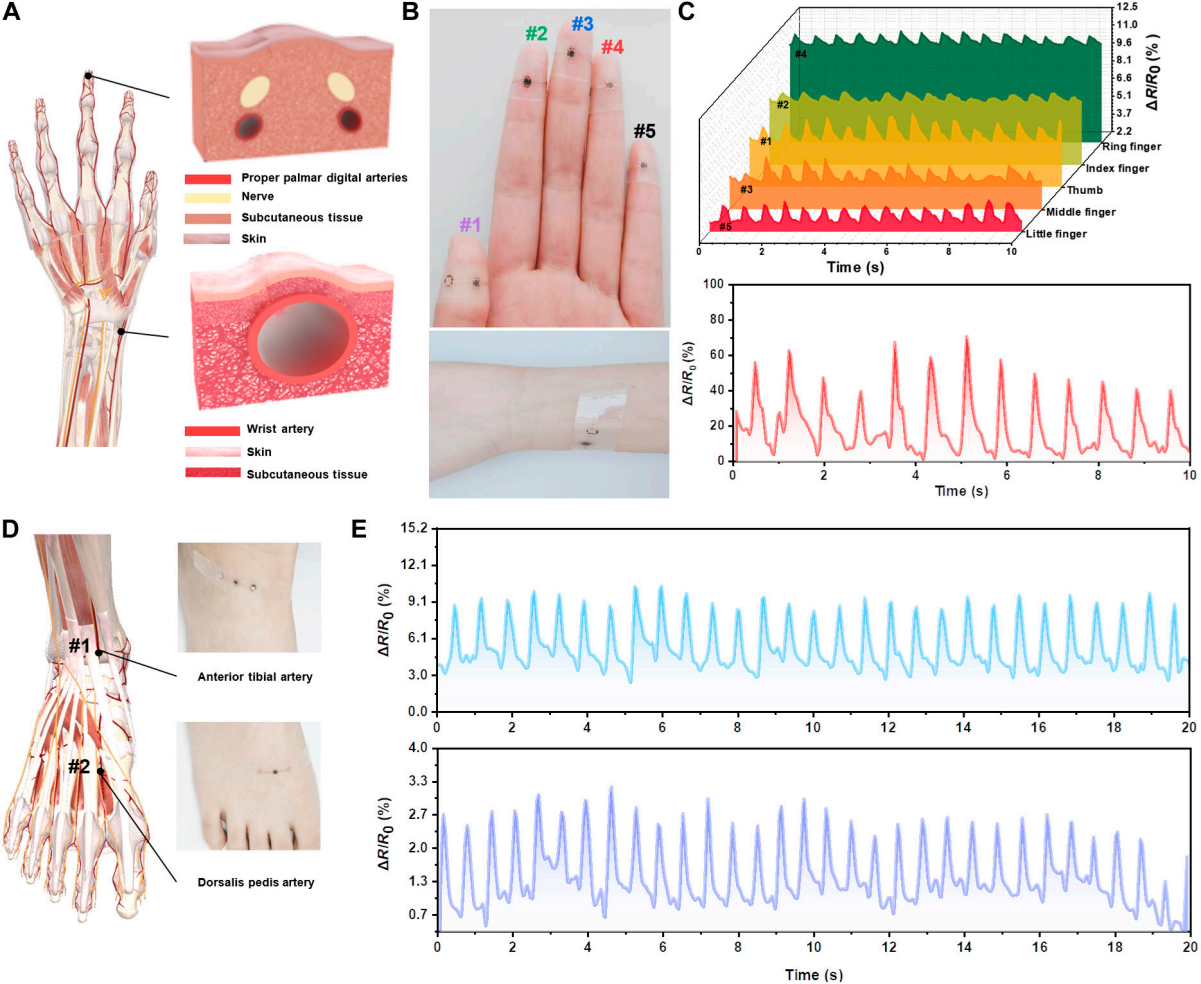
Applications of a single pressure-sensing unit. (A) Illustrations of arterial distribution on hand and a comparison of the superficial tissue artery (radial artery) and the deep tissue artery (fingertip arteries). (B) Pulse wave measurement using the sensing unit attached to the fingertips and the wrist. (C) The detected pulse waveforms at the fingertips and the wrist, respectively. (D) Distal artery pulse wave measurement using the sensor attached to the foot instep. (E) The acquired pulse waveforms at the dorsal pedal artery and the first dorsal metatarsal artery.

We apply the flexible pressure sensor to collect peripheral arterial signals. As shown in Fig. 3A, the hand is richly distributed with arterials, including the wrist artery and proper palmar digital arteries. Our sensor is firstly fixed on the wrist with polyurethane (PU) tape for radial artery pulse wave testing (Fig. 3B). The typical positive pulse waveform and characteristic peaks (percussion wave, tidal wave, and dicrotic wave) can be accurately distinguished by our sensor (Fig. 3C). More importantly, the pressure sensor can be wrapped around fingertips to sense pulse waves of the peripheral arterioles (Fig. 3B and Fig. S10A). Similar to radial pulse waves, features of the fingertip pulse waves can also be identified (Movie S1). Because of the difference in vascular diameter and depths, the amplitudes of finger signals are lower than the radial pulse. Five fingertip signals have similar pulse shapes and frequencies over a period, demonstrating the high reliability of the sensor (Fig. 3C).

We sometimes need to avoid sticking sensors on our hands, which are susceptible to manipulation and tension. Therefore, we also try to attach the sensor to foot instep to collect pulse waves. As depicted in Fig. 3D and Fig. S10B-C, sensors are stuck on the anterior tibial artery and the dorsalis pedis artery using tape, and typical pulse waves are recorded (Fig. 3E and Movie S3). Pulse signals of the anterior tibial artery are significantly higher than signals of the first metatarsal artery, the branch of the dorsal pedal artery. These results confirm that our sensor has the potential for various applications in daily healthcare monitoring.

**Fig. 4.**
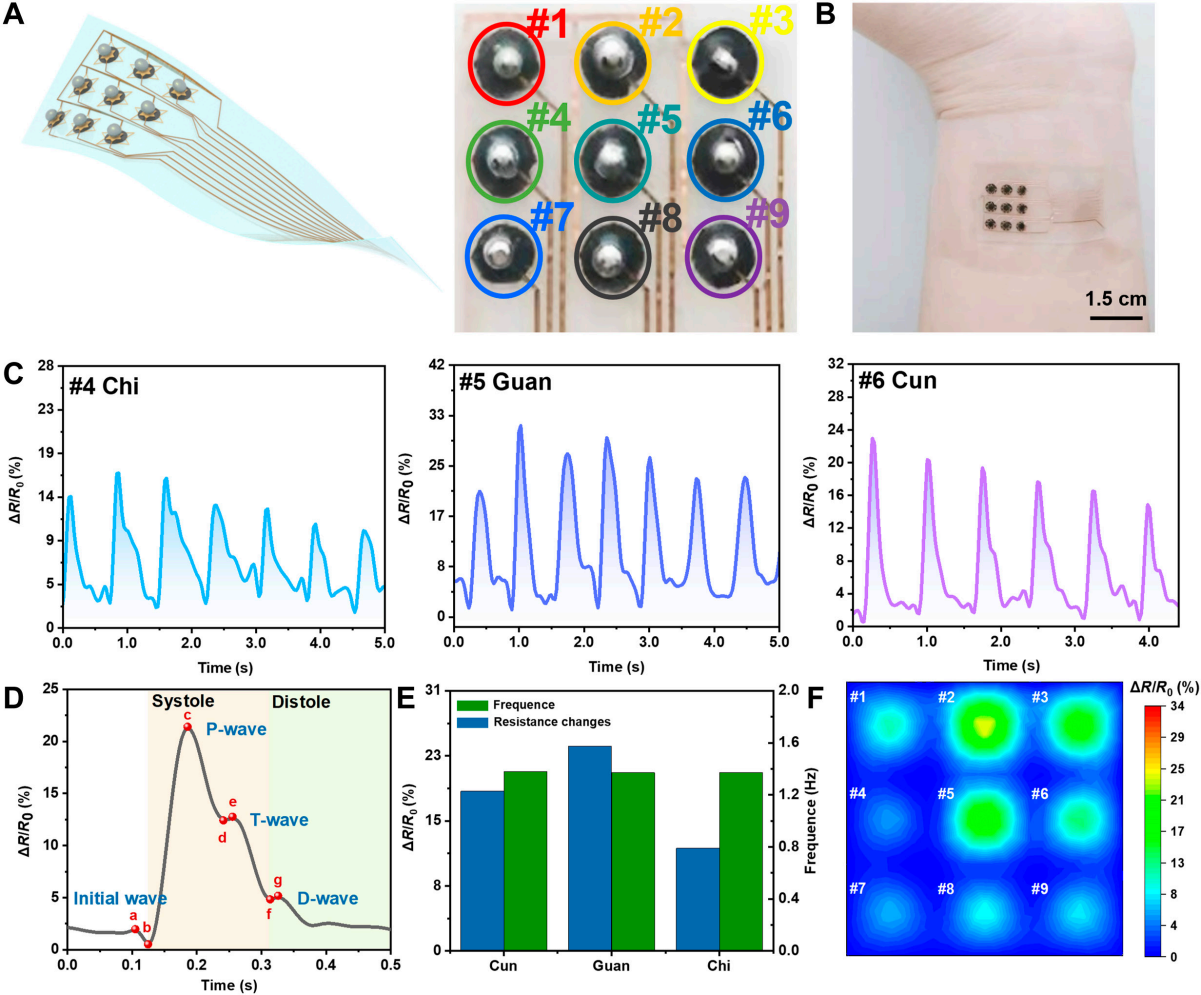
Flexible sensor array and its application for pulse condition recognition by traditional Chinese medicine. (A) Schematic illustration and photograph of a 3 × 3 pixel sensing array. (B) Photograph of the pressure sensor array at the radial artery for the pulse wave test. (C) Real-time pulse singles at “Cun,” “Guan,” and “Chi.” (D) Peak and time characteristics extracted at “Guan.” (E) Resistance change and time-frequency features at “Cun,” “Guan,” and “Chi.” (F) Three-dimensional dynamic pulse distribution.

In addition to a single element, a 3 × 3 flexible sensor array is designed (Fig. 4A) to detect pressure distribution. The detailed fabrication process is illustrated in Fig. S11-13. The 3 × 3 flexible sensing array can be stuck to the skin surface for monitoring pressure within a certain area. Here, we put the sensor array onto a fingertip to detect the actual touch, suggesting the performance on curved surface (Fig. S14). The sensor array is used to measure the pulse conductions and identify the spatial distribution of pressure (Fig. 4B). The characteristic features of the pulse conditions are acquired at “Cun,” “Guan,” and “Chi” positions (Fig. 4C). Through position calibration and distribution analysis, herbalist doctors can rapidly distinguish long pulses from short pulses. Amplitude features are extracted from average pulse waveforms over a short period, including dominating wave height, tidal wave height, dicrotic notch height, and dicrotic wave height (Fig. 4D). Intuitively, various pulse conditions, such as smooth pulses, string-like pulses, unsmooth pulses, and surging pulses, are identified and analyzed by comparing peak heights. Then, frequency features of “Cun,” “Guan,” and “Chi” are extracted by Fourier transformation and averaged to reflect heart rate (Fig. S16). Frequency features can help doctors distinguish slow pulses, rapid pulses, and irregular pulses. Combined with peak height and periods, smooth pulses, soggy pulses, and slow pulses can be further identified (Fig. 4E). According to the experimental data, the power of the peak in “Guan” is the largest, followed by “Cun” and then “Chi.” Further, as shown in Fig. 4F, the peak morphology in “Guan” is concentrated, while it is scattered in “Cun” and “Chi.” These features are highly consistent with the theory of traditional Chinese medicine [42]. On the basis of the pulse peak distribution, we acquire indirect power information around “Cun,” “Guan,” and “Chi” surroundings, which is also of great significance in analyzing the pulse shape and the pulse potential.

The judgment of pulse shape can help people monitor daily health, while calculated characteristic values assist doctors in the early diagnosis and prevention of diseases. We extract several characteristic values based on the amplitude signals of the pulse waveforms, including augmentation index (*AIX*), waveform characteristic (*K*), and blood viscosity index (*V*). In medicine, the above 3 features can be calculated by the height ratios of percussion wave (P-wave), tidal wave (T-wave), and diastolic wave (D-wave). These medical indicators can be further obtained from Eqs. 2 to 5.

$$\mathrm{AIX}=\frac{P_{c}-P_{e}}{P_{c}-P_{a}}$$ (2)

$$K=\frac{P_{m}-P_{a}}{P_{c}-P_{a}}$$ (3)

$$P_{m}=1/T\int_{0}^{T}P\left(t\right)\mathrm{dt}$$ (4)

V=11.43K (5)

where *P_c_* is the height of the P-wave, *P_e_* is the height of the T-wave, *P_a_* is the baseline value, *P_m_* is the mean artery pressure, and *T* is the pulse cycle. The aforementioned features are important cardiovascular factors that reflect arterial stiffness. The related experiments prove that *AIX* and *K* can better identify atherosclerosis in a standard reference range of 0.5 to 1.3 and 0.35 to 0.4, respectively.

Furthermore, we recruit a 26-year-old asymptomatic female volunteer to demonstrate the characteristics of hemodynamic indexes. We collect her pulse waves to calculate *AIX* and *K*. The results show that the value of *AIX* is around 0.55 and *K* is around 0.35, which are within the normal range. Then, *V* is calculated to be approximately 4.85 (standard reference range of 3.43 to 5.07). For comprehensive diagnosis, we further study 4 more valuable parameters: stroke volume (SV), cardiac output (CO), total peripheral resistance, and arterial compliance (AC). SV refers to the blood volume discharged from the left ventricle during a single cardiac stroke, which indicates the condition of cardiopulmonary function. SV is related to heart rate and the difference between systolic and diastolic blood pressure. Heart rate can be acquired by the time-frequency analysis of pulse waveforms (Fig. S16), and pulse pressure can be calibrated using a smart sphygmomanometer. The calculated average heart rate and pulse pressure are 93.15 times per minute (normal range is 60 to 100 times per minute) and 38 mmHg (normal range is 20 to 60 mmHg), respectively. The SV is 58.15 (the normal range is 50 to 80 ml). From SV, we can obtain AC. AC is usually used to predict atherosclerosis because the index changes of AC often precede the appearance of clinical symptoms. The AC index obtained in our study is about 1.195 ml/mmHg, fluctuating around the normal value of 1.5 ml/mmHg. CO is the product of SV and heart rate, which is an important indicator of cardiac ejection function for clinical research. From CO and mean arterial pressure, the peripheral resistance can be acquired, equal to the sum of all arterial resistance. CO is about 5.0 (the normal range is 4.5 to 5.5), and total peripheral resistance is approximately 1560.42 dyn·s/cm^5^, close to the normal value of 1600 dyn·s/cm^5^. Therefore, we can conclude that the calculated indexes from the measured data indicate the health conditions of the subject and provide quantitative criteria to predict early atherosclerosis.

### Blood pressure measurement

**Fig. 5.**
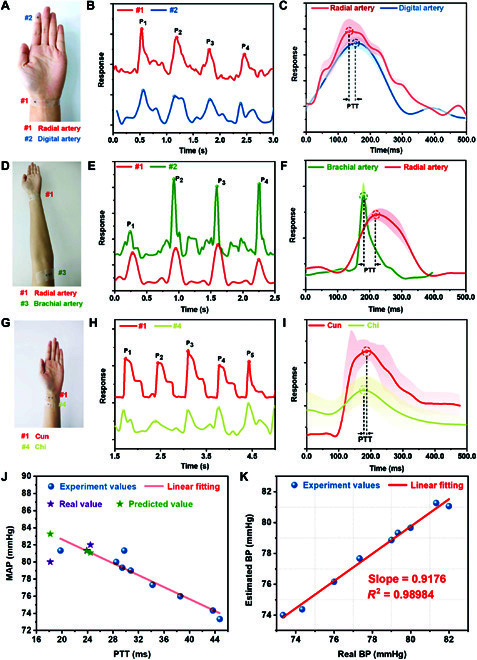
Potential application of blood pressure measurement based on the same artery branch. (A-C) Radial-volaris indicis joint pulse waveform measurement for PTT. (D-F) Radial-brachial combined pulse waveform measurement for PTT. (G-I) “Cun-Chi” associated pulse waveform measurement for PTT. (J) The linear relationship between PTT and mean arterial blood pressure (MAP). (K) The fitting curve in real blood pressure values and estimated blood pressure values.

In addition to pulse wave monitoring, the flexible pressure sensor is also capable of detecting blood pressure. The blood pressure can be obtained by measuring the pulse transit time (*PTT*) at the same artery branch (Fig. S17). By recording the arrival time of each pulse wave at various locations along the same arterial branch, a time delay (Δ*T*, defined as *PTT*) can be calculated. Then, another parameter, i.e., pulse wave velocity (*PWV*), can be obtained by the following Eq. 6:

$$\mathrm{PWV}=\frac{D_{\text{is}}}{\Delta \;T}$$ (6)

where *D_is_* is the direct surface distance of 2 pulse sites. As shown in Fig. 5A, we test *PTT* at radialis indicis artery, radial and brachial pulse sites, and “Cun” and “Chi” sites. Eight pulse waves for each pair of locations are averaged to calculate the mean *PTT*. For each pair of pulse sites, the distance is measured as 166, 245, and 25 mm, respectively. As depicted in Fig. 5B,E,H, pulse waves at different sites are simultaneously detected, and the average *PTT* is obtained (19.67, 28.5, and 4.1 ms). On the basis of Eq. 6, the averaged PWV for the 3 sites are 8.4, 8.6, and 6.1, respectively. Note that the third value is slightly lower than the other ones, which can be ascribed to the larger errors caused by a shorter distance and time difference. Considering the standard range (<9 m/s), the mean PWV indicates a low health risk for our volunteer.

In 1957, Landowne [43] proposed a linear correlation between *PTT* and arterial blood pressure within a certain range, and the relationship is relatively stable over a period. Therefore, the relationship between *PTT* and mean arterial blood pressure (*MAP*) for the same person over a short period can be described by Eq. 7:

MAP=a⋅PTT+b (7)

where *a* and *b* are undetermined parameters for the linear relationship. We utilize the cuff sphygmomanometers for this model to calibrate systolic and diastolic blood pressure and then calculate *MAP* on the basis of the data. From the linear fitting between *PTT* and *MAP*, *a* and *b* are fitted to −0.35 and 89.60 with linearity up to ~0.90 (Fig. 5J). Then, 4 pairs of *MAP* and *PTT* test values are collected to verify the accuracy of the fitting curve. The relative error is less than ±3%, which is consistent with the international association for the advancement of medical instrumentation standard. Figure 5K presents the deviations between the estimated and actual values, demonstrating the feasibility and validity of this method. The interval of 2 measurements by cuff sphygmomanometer is less than 5 min, which limits the cuff’s application in long-time blood pressure tests. Compared with a cuff sphygmomanometer, our sensor can easily estimate blood pressure without air inflation. More importantly, continuous blood pressure monitoring can be achieved for long periods.

## Discussion

In summary, we develop a novel soft-strain effect-based flexible pressure sensor in which a flexible conductive film stuck on a hollow electrode acts as a soft-strain gauge to generate and amplify tensile strain. A positive resistance variation is achieved as the applied pressure increases, which solves the problem of compression saturation as well as decreased sensitivity with increasing pressure. The proposed sensor exhibits a high sensitivity of ~26.0 kPa^−1^ in the range of 0.1 to 46 kPa, and ~636.8 kPa^−1^ in the range of 46 to 56 kPa. Moreover, high stability and durability are achieved over 10,000 cycles under 10 kPa. Further, we demonstrate that the sensor has the capability to detect weak peripheral arteriole pulses when a band is wrapped on human skin, which is difficult for most flexible pressure sensors. The flexible sensors demonstrate a capacity to rapidly locate and distinguish various pulse conditions quantitatively and establish multiple physiological indicators, such as MAP. It is expected that the flexible pressure sensor has potential in applications of wearable health monitoring for early diagnosis of cardiovascular diseases and arteriosclerosis.

## Materials and Methods

### Preparation of the copper foil electrodes and microsphere

Laser direct writing is used to write ring microelectrodes. The laser instrument is a bottom-up processing system equipped with a 1064-nm laser. Commercial copper foil with a thickness of 6 μm is used as the electrode material. The laser is directly written on the foil with designed patterns. The power of the laser is 1000 mJ, and the frequency is 20 Hz. The top electrode is designed as an openwork polygon, and the bottom electrode is a planar one. A hot-press lamination method is used to adhere the copper foil to a PDMS substrate with a thickness of 200 μm. The PDMS is purchased from the Dow Corning company. After laser writing, the desired copper electrodes are on PDMS. Then, anhydrous ethanol is used to remove the copper powder and clean the surface of the microelectrodes. Next, the top and bottom electrodes are transferred to a new PDMS film. To enhance the soft-strain effect of the pressure-sensitive layer, we align a tin microsphere with the top electrode.

### Preparation of conductive films

Conductive carbon oil is purchased from SANDOZ, Switzerland. The conductive solution is spin-coated (5000 rpm, 30 s) on PI marks and baked in an oven at 100 °C for 1 min to form pressure-sensitive films. We can also adopt a water transfer printing method to transfer the conductive film from polyvinyl alcohol to PI substrate, showing its ultrathin characteristics. The carbon film is then patterned (round shape) using a laser and assembled on the bottom electrode.

### Construction of the soft-strain pressure sensor

The top electrode is stuck on the center of the carbon conductive oil film, which forms a film on the bottom ring electrode after baking. Then, we stick a tin microsphere with Ecoflex to the top electrode to enhance the soft-strain effect. After the electrode leads are fixed, the Ecoflex solution is spin-coated for encapsulation. Consequently, strong bonds are formed between the substrate, the electrodes, and the microsphere.

### Characterization of pressure-sensing performance

Pressure-sensing performance is evaluated using a stepper pressure testing system. A stepper motor (SC100 Stepping Motor) is used to control the pressure applied to the sensing unit, and a manometer (Edberg, test range of 0 to 5 N and 0 to 50 N) is used to measure the force value. The pressure is calculated by the equation: *P* = *F*/*S* . *P* is pressure, *F* is force, and *S* is the contact area. A motor speed of 2.5 μm·s^−1^ is used for the dynamic pressure response and reliability test. A high-precision digital source meter (KEITHLEY 2450 Source Meter) and LabVIEW are connected to the electrode leads to record the resistance signals. The Young’s modulus and thickness of the PDMS substrate are 1.2 MPa and 200 μm, respectively. The thickness of copper foil electrodes is 6 μm. The radius of the bottom ring electrode and the top electrode are 500 and 200 μm, respectively. The thickness, radius, and Young’s modulus of the sensing film are 3 μm, 500 μm, and 3 MPa, respectively. Young’s modulus is tested by the material testing machine (Instaon).

### Real-time monitoring of pulse waves

PU tape is used to fix a flexible sensor on the distal and radial arteries to detect pulse waves. The external force applied by the tape is ~10 kPa. Then, the resistance signals are depicted and compared with a standard pulse wave. The materials contacted with human skin (i.e., the PU tape and PDMS) are all biologically friendly. The pulse waveform data are in original form without processing.

## Data Availability

All data needed to evaluate the conclusions in the paper are present in the paper and/or the supplementary materials.

## Supplementary Materials

Supplementary Materials Fig. S1. Structures and sensing mechanisms of typical flexible pressure sensors. Fig. S2. Finite element simulation of different pressure structures. Fig. S3. Construction process and electrode pairs. Fig. S4. The microscope side view of sensing unit as pressure increases. Fig. S5. Static mechanical properties testing. Fig. S6. Dynamic and static mechanical performance testing. Fig. S7. Sensor response to tiny pressure. Fig. S8. Thickness of conductive layer affects sensitivity. Fig. S9. Shapes of the bottom electrode affect sensitivity. Fig. S10. Pulse waveform tests on different body parts. Fig. S11. Fabrication process of array sensors. Fig. S12. Laser direct-write copper foil technology. Fig. S13. Photos of the array sensor preparation details. Fig. S14. Pressure mapping capabilities of sensor array. Fig. S15. Waveform test at “Cun,” “Guan,” and “Chi.” Fig. S16. Time-frequency signal extraction at “Cun,” “Guan,” and “Chi.” Fig. S17. The measurement of PTT based on the same arterial tree. Table S1. The comparison of flexible pressure sensors.
