# Supplementary material for: The Soft-Strain Effect Enabled High-Performance Flexible Pressure Sensor and Its Application in Monitoring Pulse Waves
Source: Research (Wash D C). 2022 Dec 15;2022:0002. doi: 10.34133/research.0002 (PMC11407520; doi:10.34133/research.0002)
Supplement: Supplementary Materials — Fig. S1. Structures and sensing mechanisms of typical flexible pressure sensors. Fig. S2. Finite element simulation of different pressure structures. Fig. S3. Construction process and electrode pairs. Fig. S4. The microscope side view of sensing unit as pressure increases. Fig. S5. Static mechanical properties testing. Fig. S6. Dynamic and static mechanical performance testing. Fig. S7. Sensor response to tiny pressure. Fig. S8. Thickness of conductive layer affects sensitivity. Fig. S9. Shapes of the bottom electrode affect sensitivity. Fig. S10. Pulse waveform tests on different body parts. Fig. S11. Fabrication process of array sensors. Fig. S12. Laser direct-write copper foil technology. Fig. S13. Photos of the array sensor preparation details. Fig. S14. Pressure mapping capabilities of sensor array. Fig. S15. Waveform test at “Cun,” “Guan,” and “Chi.” Fig. S16. Time-frequency signal extraction at “Cun,” “Guan,” and “Chi.” Fig. S17. The measurement of PTT based on the same arterial tree. Table S1. The comparison of flexible pressure sensors. [file 0002.f1.docx]

# Supplementary Information for

# The Soft-Strain Effect Enabled High-Performance Flexible Pressure Sensor and Its Application in Monitoring Pulse Waves

## Yue Li^1,2,7^, Yuan Wei^1,2,3,7^, Yabao Yang^1,2^, Lu Zheng^1,2,3^, Lei Luo^1,2^, Jiuwei Gao^1,2^, Hanjun Jang^1,2^, Juncai Song^1,2^, Manzhang Xu^1,2,3^, Xuewen Wang^1,2,3,4*^, Wei Huang^1,2,3,5,6*^

1, Frontiers Science Center for Flexible Electronics (FSCFE) & Institute of Flexible Electronics (IFE), Northwestern Polytechnical University, 127 West Youyi Road, Xi’an, 710072, China.

2, MIIT Key Laboratory of Flexible Electronics (KLoFE), Northwestern Polytechnical University, 127 West Youyi Road, Xi’an, 710072, China.

3, Shaanxi Key Laboratory of Flexible Electronics (KLoFE), Northwestern Polytechnical University, Xi’an, 710072, China.

4, Key laboratory of Flexible Electronics of Zhejiang Provience, Ningbo Institute of Northwestern Polytechnical University, 218 Qingyi Road, Ningbo, 315103, China.

5, State Key Laboratory of Organic Electronics and Information Displays, Institute of Advanced Materials (IAM), Nanjing University of Posts & Telecommunications, Nanjing, 210023, China.

6, Key Laboratory of Flexible Electronics（KLoFE）and Institute of Advanced Materials (IAM), Nanjing Tech University (NanjingTech), Nanjing, 211800, China.

7, Yue Li and Yuan Wei contributed equally to this work

*Corresponding author.

Prof. Xuewen Wang, Email: iamxwwang@nwpu.edu.cn

Prof. Wei Huang, E-mail: iamwhuang@nwpu.edu.cn

**Table of Contents**

**SENSING MECHANISMS AND STRUCTURES.** **. 3**

[Figure S1 Structures and sensing mechanisms of typical flexible pressure sensors. 3](#_Toc80906475)

[Figure S2 Finite element simulation of different pressure structures. 4](file:///G:\2021\论文\图\Supporting%20Information.docx#_Toc80906476)

[Figure S3 Construction process and electrode pairs. 5](file:///G:\2021\论文\图\Supporting%20Information.docx#_Toc80906477)

[Figure S4 The microscope side view of sensing unit as pressure increases. 6](file:///G:\2021\论文\图\Supporting%20Information.docx#_Toc80906478)

**CHARACTERIZATIONS OF THE SENSOR..............................................................................7**

[Figure S5 Static mechanical properties testing. 7](file:///G:\2021\论文\图\Supporting%20Information.docx#_Toc80906479)

[Figure S6 Dynamic and static mechanical performance testing. 8](file:///G:\2021\论文\图\Supporting%20Information.docx#_Toc80906480)

[Figure S7 Sensor response to tiny pressure. 9](file:///G:\2021\论文\图\Supporting%20Information.docx#_Toc80906481)

Figure S8 Thickness of conductive layer affects sensitivity. 10

Figure S9 Shapes of the bottom electrode affects sensitivity. 11

[Table S1 The comparison of flexible pressure sensors. 12](#_Toc80906482)

[Figure S10 Pulse waveform tests on different body parts. 13](file:///G:\2021\论文\图\Supporting%20Information.docx#_Toc80906483)

**FABRICATION METHOD AND ITS APPLICATIONS...........................................................14**

[Figure S11 Fabrication process of array sensors. 14](file:///G:\2021\论文\图\Supporting%20Information.docx#_Toc80906484)

[Figure S12 Laser direct write copper foil technology. 15](file:///G:\2021\论文\图\Supporting%20Information.docx#_Toc80906485)

[Figure S13 The photos of the array sensor preparation details. 16](file:///G:\2021\论文\图\Supporting%20Information.docx#_Toc80906486)

[Figure S14 Pressure mapping capabilities of sensor array .17](file:///G:\2021\论文\图\Supporting%20Information.docx#_Toc80906487)

[Figure S15 Waveforms test at “Cun”, “Guan” and “Chi”. 18](file:///G:\2021\论文\图\Supporting%20Information.docx#_Toc80906488)

[Figure S16 Time-frequency signal extraction at “Cun”, “Guan” and “Chi”. 19](file:///G:\2021\论文\图\Supporting%20Information.docx#_Toc80906489)

[Figure S17 The measurement of PTT based on the same arterial tree. 20](#_Toc80906490)

**REFERENCE................................................................................................................21**


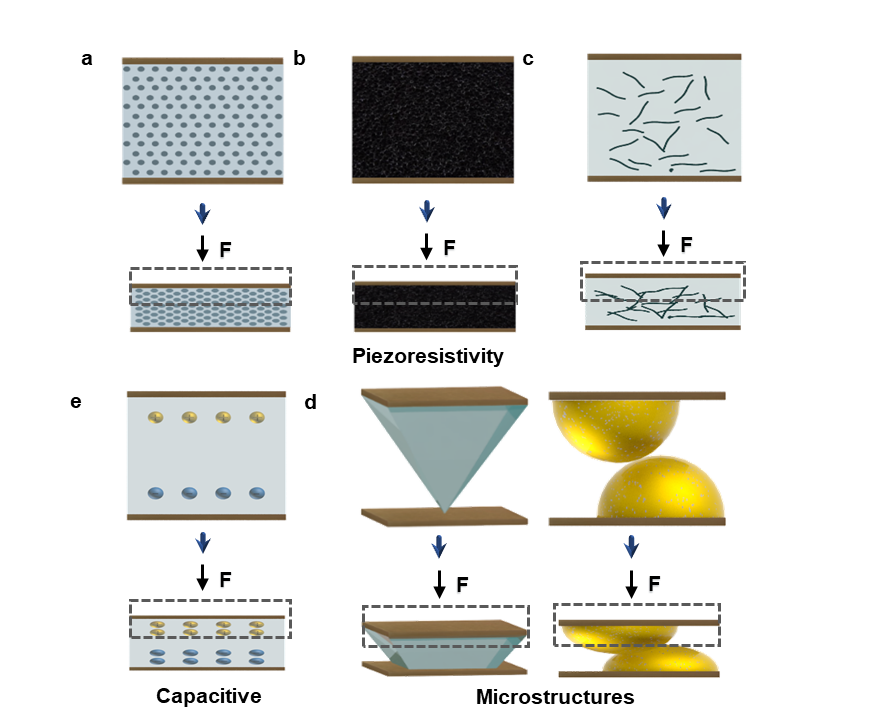


Figure S1 Structures and sensing mechanisms of typical flexible pressure sensors. a-c) Sensors based on the tunneling effect, piezoresistance, and conductive paths. d) The improvement of sensing performance by integrating microstructures. e) Sensors based on capacitance.


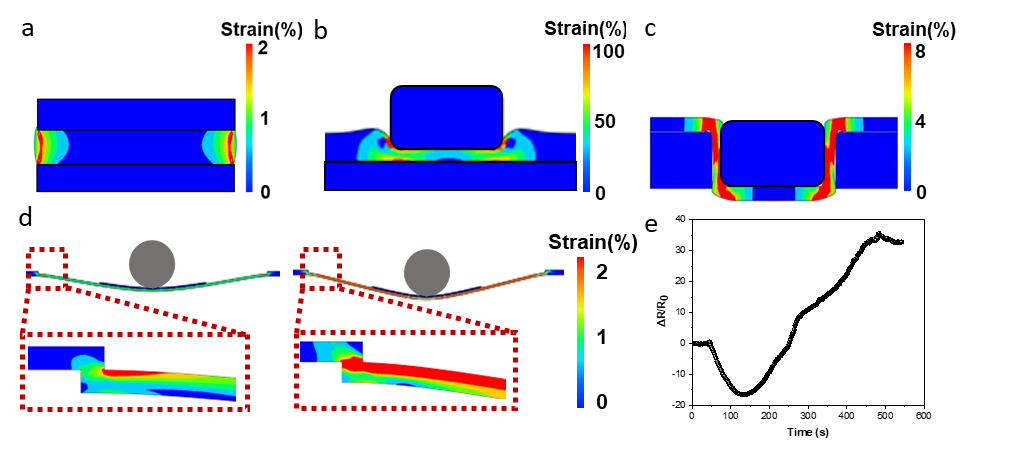


Figure S2 Finite element simulation of different pressure structures. a) Strain simulation of “sandwich” piezoresistive structures. b) Strain simulation of piezoresistive structure with asymmetric electrode pairs. c) Strain simulation of the strain effect structure. d) Simulation details of the strain effect structure as pressure increases. e) The changes in the resistance of structure b).


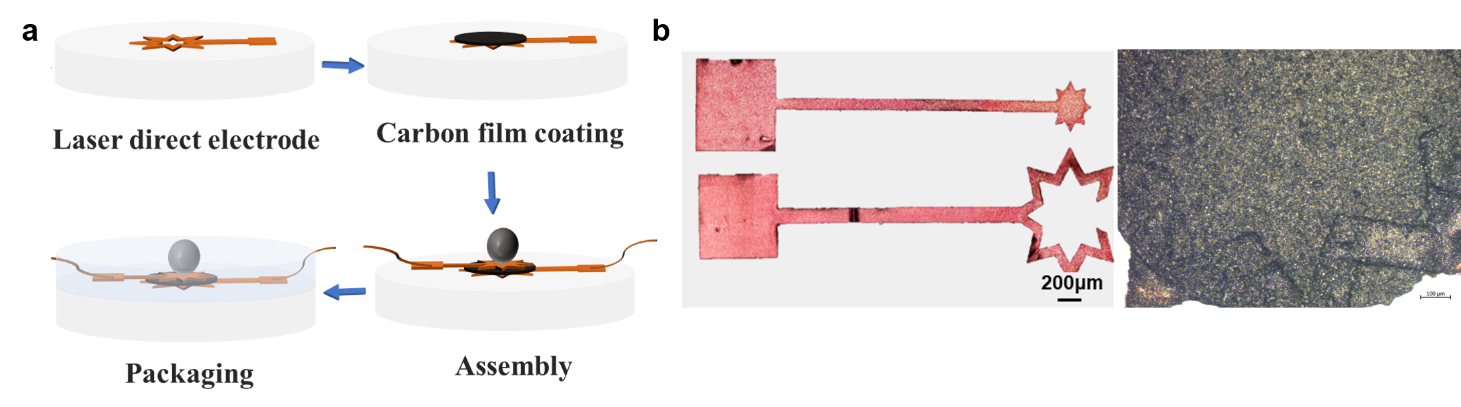


**Figure S3 Construction process of electrode pairs.** a) Laser direct writing and template method are used to construct the soft-strain effect-based pressure sensing unit, which is beneficial to fabricate array sensors. b) Details of electrode pairs made by laser direct writing.And The conductive film and bottom electrode are bond together perfectly.


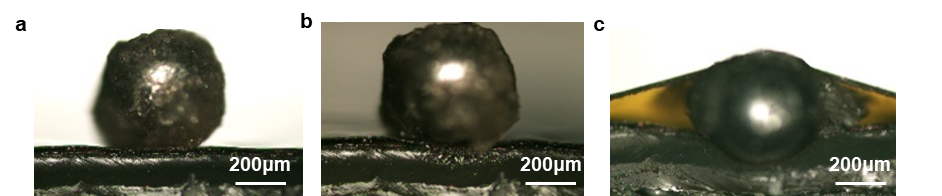


Figure S4 The microscope side view of sensing unit as pressure increases. a) The side view without pressure. b) The side view under 0.55 N. c) The side view under 1.5 N.


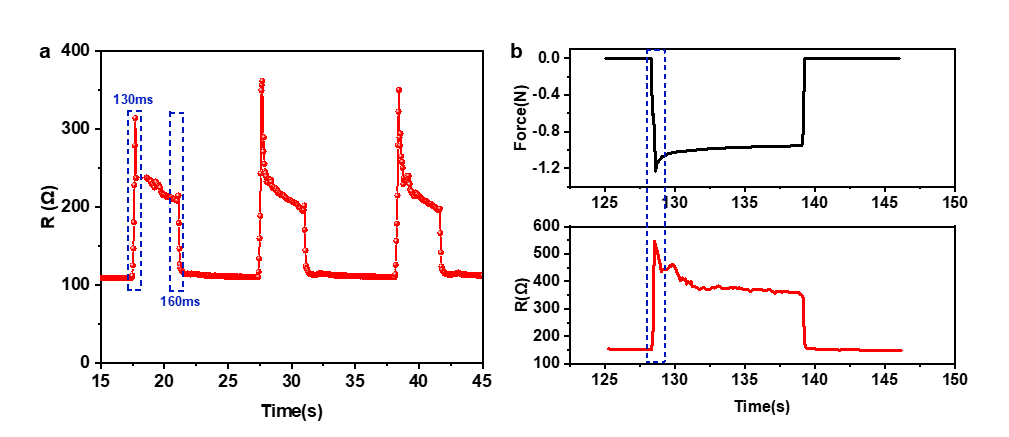


Figure S5 Static mechanical properties. a) Response time of the pressure sensor. b) Shoulder peak of the resistance corresponds to the mechanical shoulder peak, indicating the high sensitivity of the sensor.


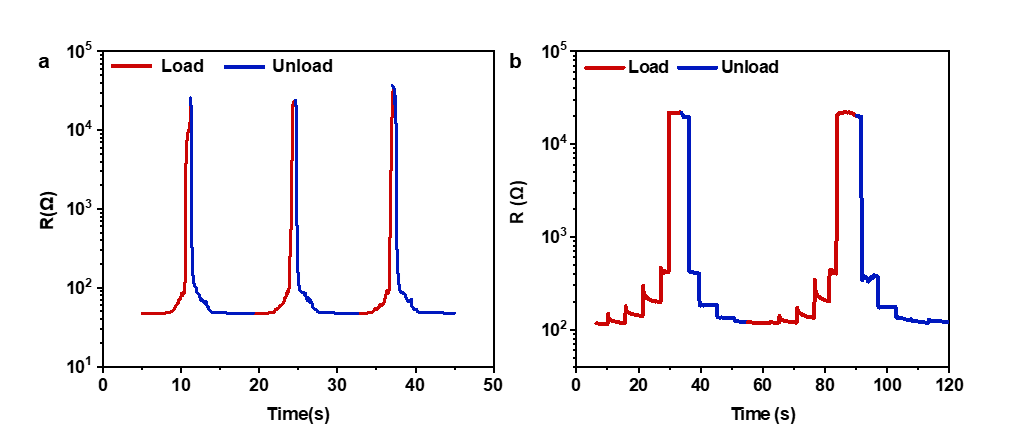


# Figure S6 Dynamic and static mechanical performance test.


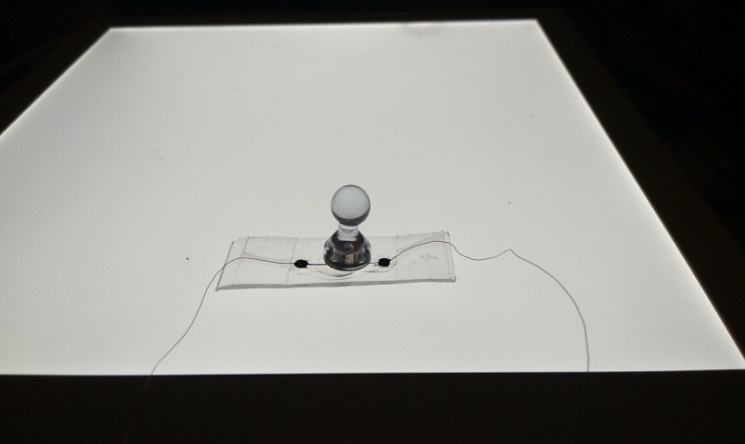


# Figure S7 Sensor response to tiny pressure (0.3 kPa).

# Figure S8 Thickness of the conductive layer affects sensitivity. The resistance change under different pressures for the sensors with a conductive film thickness of 3 and 12 μm.

**Figure S9 Shapes of the bottom electrode affects sensitivity.** The resistance change under different pressures for the sensors with various shapes of the bottom electrode, including circle, four-angle star, and anise star.

Table S1 Comparison of flexible pressure sensors.

| Low-Pressure Range | S（kPa^-1^） | High-Pressure Range | S（kPa^-1^） | Ref |
| --- | --- | --- | --- | --- |
| < 5 kPa | -1.14 | 5-50 kPa | <-0.8 | **1** |
| < 0.1 kPa | -5.53 | 0.1-1.4 kPa | -0.01 | **2** |
| 0-100 kPa | -0.025 | - | - | **3** |
| < 0.2 kPa | -75-121 | 0.2-10 kPa | >-15 | **4** |
| 0-0.2 kPa | -2 | 0.2-2.5 kPa | -0.02 | **5** |
| < 0.6 kPa | -23 | 0.6-3 kPa | -0.7 | **6** |
| < 50 kPa | -5.66 🞨10^-3^ | 50-3000 kPa | -0.23🞨10^-3^ | **7** |
| 0.0007-160 kPa | -1.02 | - | - | **8** |
| 0-12 kPa | -8.5 | - | - | **9** |
| 0-0.2 kPa | -136.8 | 0.2-1kPa | -3.75 | **10** |
| 0-0.1 kPa | < -4.196 | 0.1-0.5 kPa | ~-2.5 | **11** |
| 0.2-25 kPa | -1.2 | - | - | **12** |
| < 4.7 kPa | -151.4 | 4.7-15 kPa | -33.8 | **13** |
| < 20 kPa | -1875.5 | 20-40 kPa | -853.2 | **14** |
| < 0.1 kPa | -71.37 | 0.1-1 kPa | -2.88 | **15** |
| < 0.5 kPa | -0.8763 | 0.5 – 1 kPa | -0.411 | **16** |
| < 3.5 kPa | -0.141 | 3.5 – 10 kPa | -0.048 | **17** |
| < 0.04 kPa | 6.258 | 0.04 – 1 kPa | -4.169 | **18** |
| 1-10.3 kPa | -0.03738 | 10.3 – 204.7 kPa | -0.00059 | **18** |
| < 0.25 kPa | -514 | 0.25-1.5 | -32 | **19** |
| < 250 kPa | -15.4 | 250 – 300 kPa | ~-1 | **20** |


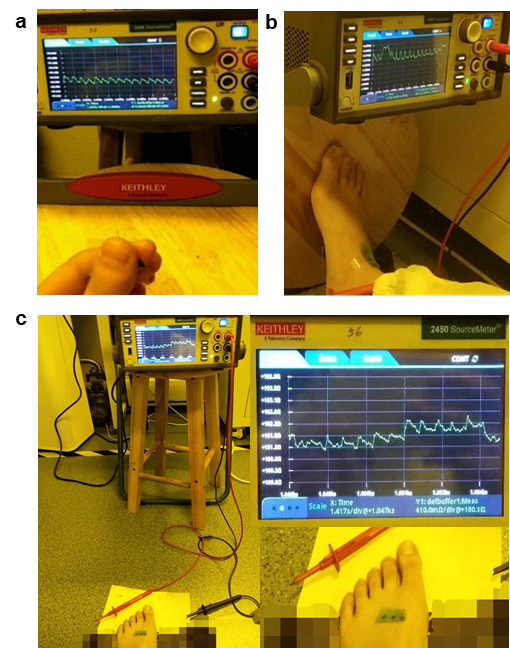


Figure S10 Pulse waveform tests on different body parts. a) The digital artery pulse waveform test on fingertips. b) The dorsal pedal artery pulse waveform test on foot. c) The first dorsal metatarsal artery pulse waveform test on foot.


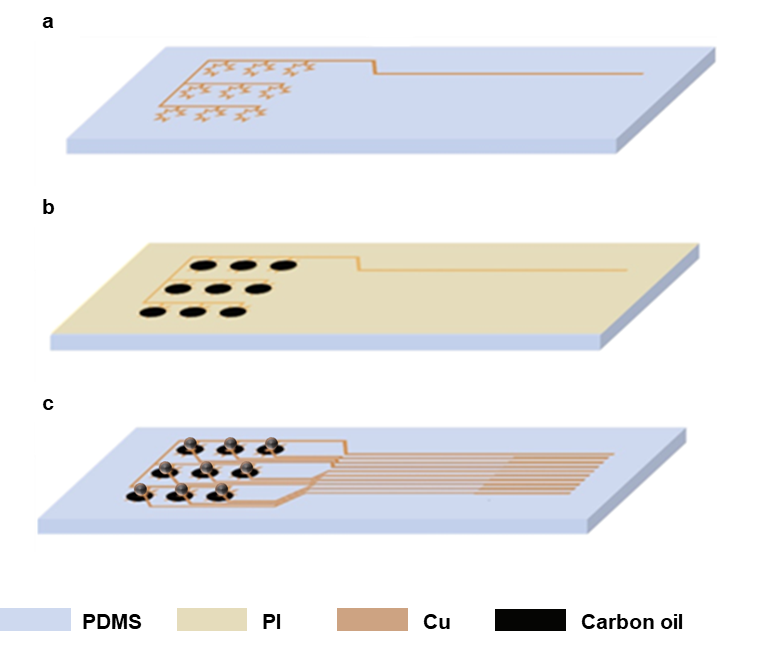


Figure S11 Fabrication process of array sensors. a) Laser direct writing on copper foil to make electrode pairs. b) Spin coat conductive carbon oil on ring bottom electrode by template method. c) Lap the top electrode when carbon oil semi-solidified to make microspheres stick on top electrode, and then encapsulate the strain effect structure.


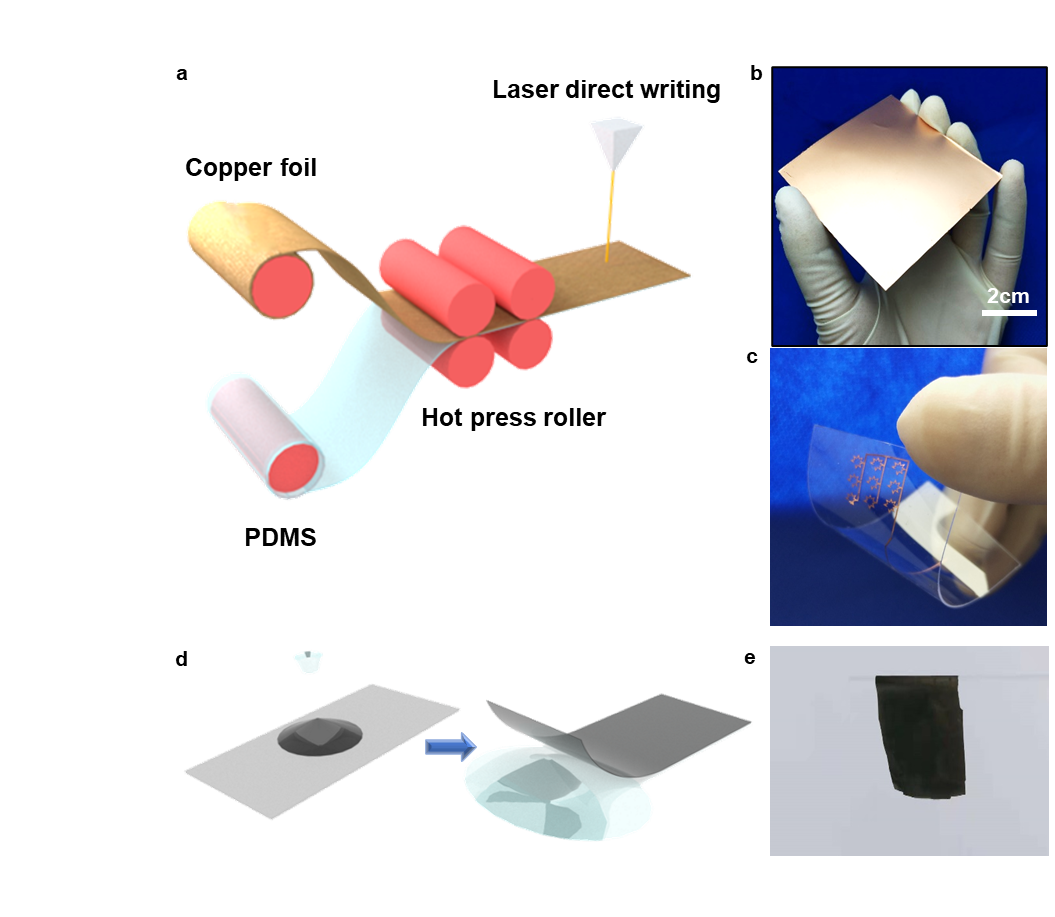


Figure S12 Laser direct writing copper foil. a) Hot press copper foil on PDMS substrate and laser direct writing the electrodes. b) Large-area copper/PDMS substrate. c) Flexible copper foil electrode fabricated by laser direct writing. d) Spin-coating and water transfer printing of conductive film. e) Photograph of the prepared conductive film.


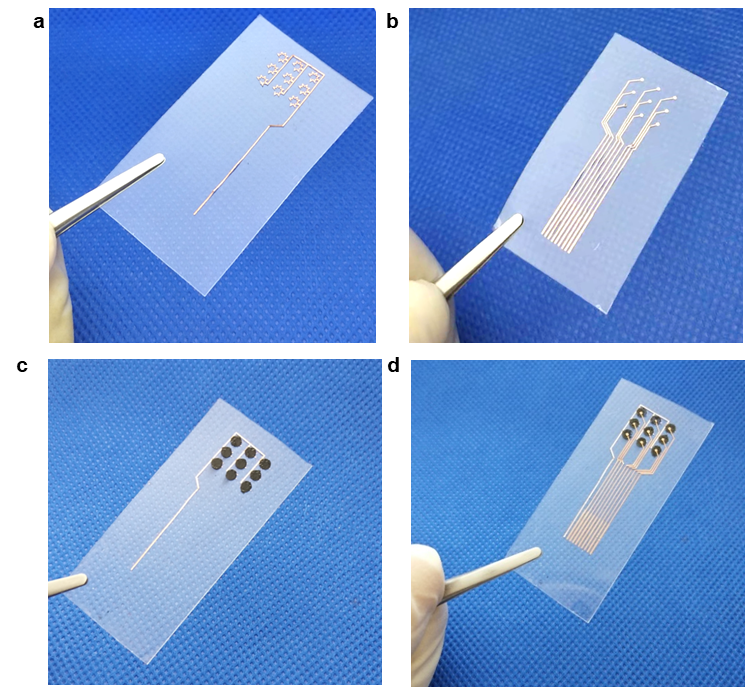


Figure S13 Photographs of the sensor array. a-b) The bottom and top electrode was written by laser direct writing technique. c) Sensing film was lapped on the bottom electrode. d) The top electrode and microspheres were aligned and lapped on the sensing film.


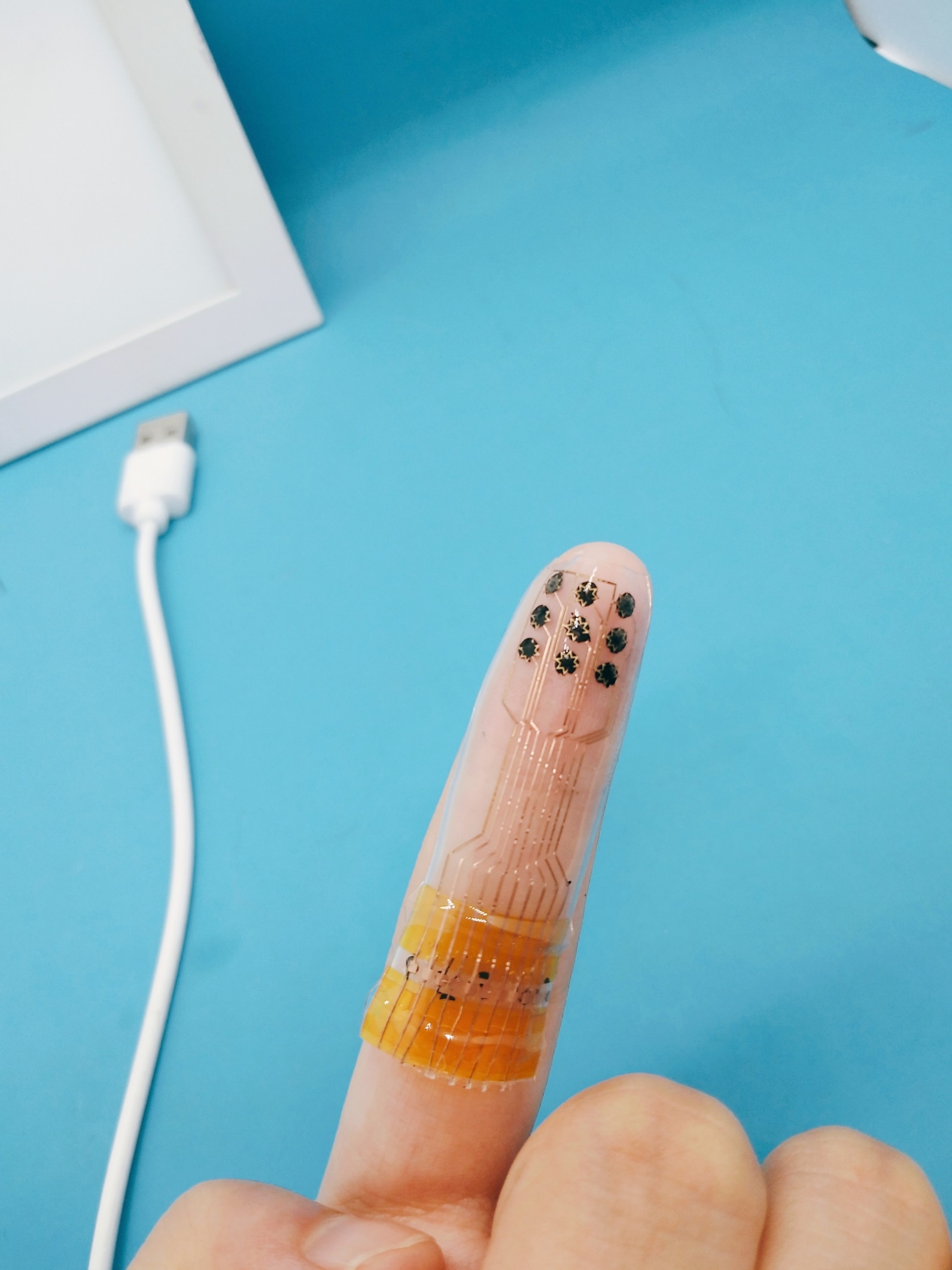


**a**

**b**

# Figure S14 Pressure mapping capabilities of sensor array and real-time touch measurement.


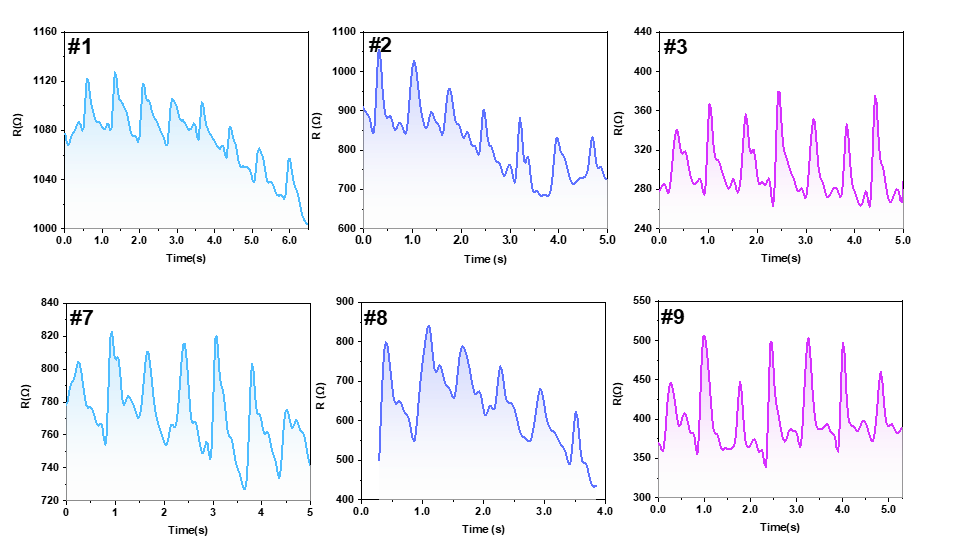


Figure S15 Waveforms test at “Cun”, “Guan” and “Chi”. The radial artery pulse waveform measurements by 3x3 array sensor at “Cun”, “Guan” and “Chi”, corresponding to the positions in Figure 4b.


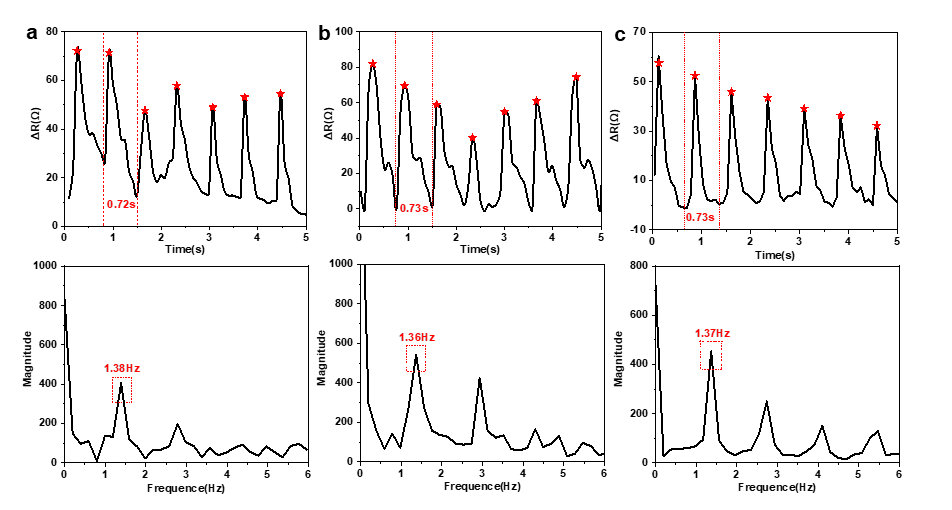


Figure S16 Time-frequency signal extraction at “Cun”, “Guan” and “Chi”. The signals are corresponding to position “#6”, “#5” and “#4”.


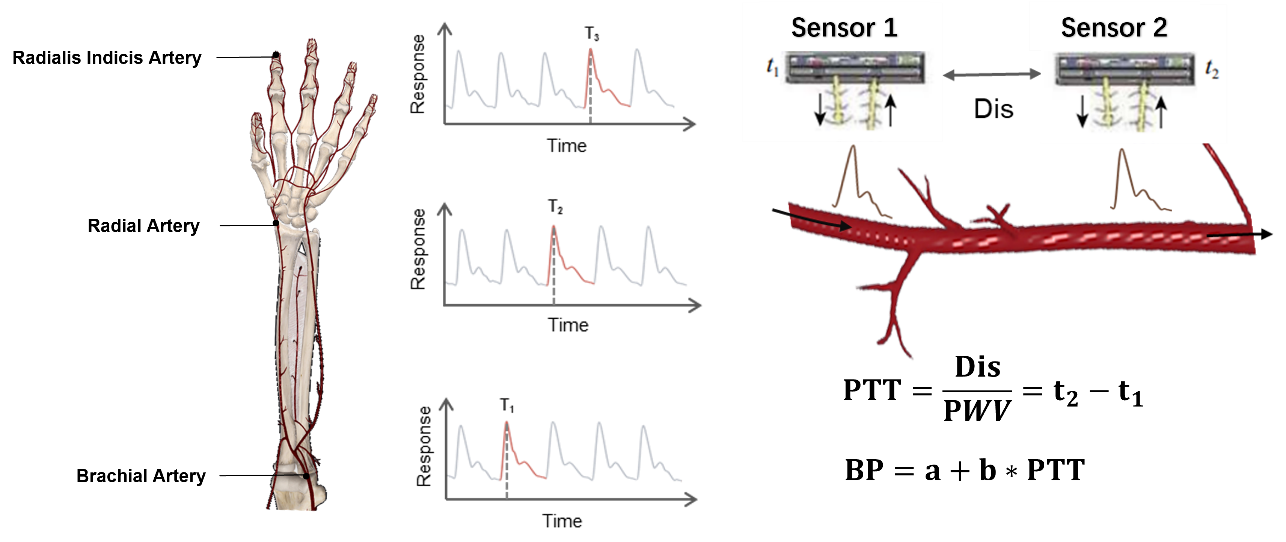


Figure S17 The PTT measurement based on the same arterial branch. The principle of measuring pulse wave time difference between the brachial artery, radial artery, and volaris indicis radialis.

**REFERENCE**

1 Gong, S. *et al.* A Wearable and Highly Sensitive Pressure Sensor with Ultrathin Gold Nanowires. *Nat. Commun.* 5, 3132 (2014).

2 Zhu, B. *et al.* Microstructured Graphene Arrays for Highly Sensitive Flexible Tactile Sensors. *Small* 10, 3625 (2014).

3 Jung, S. *et al.* Reverse-Micelle-Induced Porous Pressure-Sensitive Rubber for Wearable Human–Machine Interfaces. *Adv. Mater.* 26, 4825 (2014).

4 Yin, B. et al. Bioinspired and Bristled Microparticles for Ultrasensitive Pressure and Strain Sensors. *Nat. Commun.* 9, 5161 (2018).

5 Zhu, Y. *et al.* Highly Sensitive and Skin-like Pressure Sensor Based on Asymmetric Double-layered Structures of Reduced Graphite Oxide. *Sens. Actuators B Chem.* 255, 1262 (2018).

6 Zhu, B. *et al.* Hierarchically Structured Vertical Gold Nanowire Array-Based Wearable Pressure Sensors for Wireless Health Monitoring. *ACS Appl. Mater. Interfaces* 11, 29014 (2019).

7 Jeong, Y. *et al.* Ultra-Wide Range Pressure Sensor Based on a Microstructured Conductive Nanocomposite for Wearable Workout Monitoring. *Adv. Healthc. Mater.* 10, 2001461 (2021).

8 Yin, Y. M. *et al.* Facile Fabrication of Flexible Pressure Sensor with Programmable Lattice Structure. *ACS Appl. Mater. Interfaces* 13, 10388 (2021).

9 Bae, G. Y. *et al.* Linearly and Highly Pressure-Sensitive Electronic Skin Based on a Bioinspired Hierarchical Structural Array. *Adv. Mater.* 28, 5300-5306 (2016).

10 Zhao, T. *et al.* Highly Sensitive Flexible Piezoresistive Pressure Sensor Developed Using Biomimetically Textured Porous Materials. *ACS Appl. Mater. Interfaces* 11, 29466 (2019).

11 Xia, T. *et al.* Ultrahigh Sensitivity Flexible Pressure Sensors Based on 3D-Printed Hollow Microstructures for Electronic Skins. *Adv. Mater. Technol.* 6, 2000984 (2021).

12 Shi, J. *et al.* Multiscale Hierarchical Design of a Flexible Piezoresistive Pressure Sensor with High Sensitivity and Wide Linearity Range. *Small* 14, 1800819 (2018).

13 Cheng, Y. *et al.* Bioinspired Microspines for a High-Performance Spray Ti3C2Tx MXene-Based Piezoresistive Sensor. *ACS Nano* 14, 2145 (2020).

14 He, J. *et al.* A Universal High Accuracy Wearable Pulse Monitoring System via High Sensitivity and Large Linearity Graphene Pressure Sensor. *Nano Energy* 59, 422 (2019).

15 Lee, D. *et al.* High-performance Transparent Pressure Sensors Based on Sea-urchin Shaped Metal Nanoparticles and Polyurethane Microdome Arrays for Real-time Monitoring. *Nanoscale* 10, 18812 (2018).

16 He, Y., Zhao, L., Wang, X., Liu, L. & Liu, H. Microstructured Hybrid Nanocomposite Flexible Piezoresistive Sensor and Its Sensitivity Analysis by Mechanical Finite-Element Simulation. *Nanotechnology* 31, 185502 (2020).

17 Dan, L., Shi, S., Chung, H.-J. & Elias, A. Porous Polydimethylsiloxane–Silver Nanowire Devices for Wearable Pressure Sensors. *ACS Appl. Nano Mater.* 2, 4869 (2019).

18 Zhu, G.-J. *et al.* A Highly Sensitive and Broad-Range Pressure Sensor Based on Polyurethane Mesodome Arrays Embedded with Silver Nanowires. *ACS Appl. Mater. Interfaces* 12, 19988 (2020).

19 Wang, Z. *et al.* The Semiconductor/Conductor Interface Piezoresistive Effect in an Organic Transistor for Highly Sensitive Pressure Sensors. *Adv. Mater.* 31, 1805630 (2019).

20 Li, Z., Zhang, B., Li, K., Zhang, T. & Yang, X. A Wide Linearity Range and High Sensitivity Flexible Pressure Sensor with Hierarchical Microstructures via Laser Marking. *J. Mater. Chem. C* 8, 3088 (2020).
